# 2025 Brazilian evidence-based guideline on the management of obesity and prevention of cardiovascular disease and obesity-associated complications: a position statement by five medical societies

**DOI:** 10.1186/s13098-025-01954-8

**Published:** 2025-11-18

**Authors:** Cynthia M. Valerio, Jose F. K. Saraiva, Fernando Valente, Simone van de Sande-Lee, Viviane Z. Rocha, Fabiana H. Rached, Luciano F. Drager, Bruno Halpern, Wellington S. Silva Júnior, Fabio R. Trujilho, Neuton Dornelas, Ruy Lyra da Silva Filho, João Eduardo N. Salles, Marcelo H. V. Assad, Marcio C. Mancini, Paulo A. C. Miranda, Rodrigo Moreira, Rodrigo N. Lamounier, Sergio Kaiser, Marcello C. Bertoluci

**Affiliations:** 1 Instituto Estadual de Diabetes E Endocrinologia Luiz Capriglione (IEDE-RJ), Department of Metabolism, Rio de Janeiro-RJ, Brazil; 2 Disciplina de Cardiologia da Pontifícia, Universidade Católica de, Campinas- SP, Brazil; 3 Faculdade de Medicina Do ABC-SP, Santo André, Brazil; 4 Universidade Federal de Santa Catarina, Florianópolis-SC, Brazil; 5 Instituto Do Coração (InCor) da Faculdade de Medicina da Universidade de, São Paulo (FMUSP)- SP, Brazil; 6 Centro de Controle de Peso Do Hospital 9 de Julho, São Paulo, Brasil; 7 Universidade Federal Do Maranhão (UFMA), São Luís – MA, Brazil; 8 Instituição Centro de Diabetes E Endocrinologia da Bahia (CEDEBA), Salvador-BA, Brazil; 9 Hospital da Obesidade, Salvador-BA, Brazil; 10 Corpo de Bombeiros Militar Do Distrito, Federal-DF, Brazil; 11 Universidade Federal de, Recife, Pernambuco Brazil; 12 Faculdade de Ciências Médicas da Santa Casa de, São Paulo-SP, Brazil; 13 Instituto Nacional de Cardiologia –Rio de Janeiro, Rio de Janeiro, Brazil; 14 Unidade de Obesidade da Disciplina de Endocrinologia E Metabologia Do, HCFMUSP, São Paulo-SP, Brazil; 15 Santa Casa de , Belo Horizonte-MG, Brazil; 16 Rede Mater Dei de Saúde, Belo Horizonte-MG, Brazil; 17 Centro Universitário Presidente Antônio Carlos (UNIPAC), Juiz de Fora-MG, Brazil; 18 Disciplina de Clínica Médica, Faculdade de Medicina, UFMG, Belo Horizonte, Minas Gerais Brazil; 19 Universidade Estadual Do Rio de Janeiro (UERJ), Rio de Janeiro-RJ, Brazil; 20 Serviço de Endocrinologia, Hospital de Clínicas de Porto Alegre, Porto Alegre, Rio Grande Do Sul Brazil; 21 Faculdade de Medicina da, Universidade Federal Do Rio Grande Do Sul, Porto Alegre, Rio Grande Do Sul Brazil

**Keywords:** Obesity, Anti-obesity treatment, Cardiovascular disease, Heart failure, Guidelines, Obesity-related complications

## Abstract

**Background:**

Obesity, which currently affects over one billion individuals, is widely recognised as a global condition. It is strongly associated with an increased incidence of cardiovascular disease (CVD), which accounts for 26.8% of all deaths worldwide. The emergence of new anti-obesity medications that can provide greater weight loss and more significant clinical benefits has underscored the urgent need for structured guidelines that integrate obesity treatment into CVD prevention strategies. This article, developed through a collaboration among five leading Brazilian medical societies (Brazilian Association for the Study of Obesity and Metabolic Syndrome, Brazilian Diabetes Society, Brazilian Society of Endocrinology and Metabolism, Brazilian Cardiology Society, and Brazilian Sleep Academy), aims to structure obesity treatment within the context of CVD prevention, considering both cardiovascular risk and obesity stage.

**Methods:**

The Delphi method was used to develop the guideline by engaging a panel of twenty experts who formulated 25 evidence-based recommendations through multiple rounds of structured voting. Each recommendation was designed to address specific clinical scenarios and assigned a recommendation grade based on statistical analysis consensus levels.

**Results:**

Following cardiovascular risk assessment using the Predicting Risk of CVD Events risk score, individuals with obesity or overweight will be stratified according to their 10-year risk of developing atherosclerotic disease (low, moderate, or high) and heart failure (high-risk). Anti-obesity treatment will then be guided by the best evidence-based recommendations designed to address excess adiposity and reduce associated complications.

**Conclusion:**

This guideline offers a practical, evidence-based framework for the treatment of obesity, primarily focusing on the prevention of obesity-related complications, particularly CVD. By applying these recommendations, healthcare professionals can tailor therapeutic strategies to the specific needs of individuals living with obesity. We hope that the widespread implementation of this guideline will contribute to reducing the adverse health burden of obesity and CVD, improving public health outcomes in Brazil.

**Supplementary Information:**

The online version contains supplementary material available at 10.1186/s13098-025-01954-8.

## Introduction

Global obesity prevalence has nearly tripled since 1975, now affecting over one billion people. Obesity is widely recognised as a condition associated with numerous chronic diseases, significantly impairing quality of life and reducing life expectancy [1].

In 2021, 612 million individuals were affected by cardiovascular disease (CVD), accounting for 26.8% of all deaths worldwide. This figure has increased by 0.88% over the past 30 years. Notably, 79.5% of all disability-adjusted life years (DALYs) lost can be attributed to 11 risk factors, with body mass index (BMI) showing the strongest association [2]. Furthermore, prevalence studies have shown that approximately two-thirds of obesity-related deaths are due to CVD [2, 3]. The Brazilian data from 2025 show that 68% of adults have a BMI of at least 25 kg/m^2^, with 31% classified as living with obesity. In 2021 alone, 60,913 premature deaths in Brazil were attributed to elevated BMI [1].

The relationship between obesity and CVD is well-established. Prospective epidemiological studies have shown that obesity increases the risk of coronary artery disease (CAD) events and cardiovascular mortality [4]. Obesity contributes to the development of CVD through multiple pathways, either indirectly through increased traditional cardiovascular (CV) risk factors, such as type 2 diabetes, dyslipidaemia, and hypertension, or directly through an adiposity-induced inflammatory state that affects cardiac structure and function [5, 6].

Multiple epidemiological studies have related obesity to CVD through BMI. A meta-analysis of over 300,000 adults showed that BMI-defined overweight and obesity ranges are associated with increased risk of CAD and CV mortality. Observational and Mendelian randomisation studies have indicated a strong direct link between higher BMI and increased heart failure incidence and mortality [7].

In addition, abdominal obesity is reported as more directly associated with increased risk of cerebrovascular disease, coronary heart disease, and CV mortality [5]. Meta-analyses of large cohort studies showed that abdominal obesity, measured by waist circumference, is a strong independent predictor of morbidity and mortality across all BMI categories [5, 6]. Even individuals with a BMI below 30 kg/m^2^ may present with elevated cardiometabolic risk, particularly when visceral fat accumulation is accompanied by a relative deficiency in gluteofemoral subcutaneous fat and other risk factors [8]. Therefore, alternative measurements to BMI, such as waist circumference, waist-to-hip ratio, and waist-to-height ratio are recommended to better identify individuals with potential visceral adiposity [9–11].

Despite the availability of various treatment options, including lifestyle interventions, pharmacotherapy, and bariatric surgery, the management of obesity remains challenging. Achieving and maintaining weight loss can be challenging, and the long-term outcomes of obesity treatment are frequently modest. Moreover, the increasing complexity of anti-obesity treatments, some with proven benefits in cardiorenal-metabolic syndrome outcomes, underscores the urgent need for new stratification tools to guide treatment selection in specific clinical situations. [12, 13].

Accordingly, this guideline aims to structure obesity treatment within the context of CVD prevention, considering both CV risk and obesity stage. It provides evidence-based recommendations to support healthcare professionals in personalizing optimal therapeutic strategies for individuals living with obesity.

## Methodology

The guideline was developed using the Delphi method [14], a structured process involving successive rounds of expert input, in which participants respond anonymously and are afforded opportunities to revise their responses based on feedback from other participants.

An initial panel of twenty experts from five medical societies, Brazilian Association for the Study of Obesity and Metabolic Syndrome (ABESO), Brazilian Diabetes Society (SBD), Brazilian Society of Endocrinology and Metabolism (SBEM), Brazilian Cardiology Society (SBC), and Brazilian Sleep Academy (ABS), were recruited. From this panel, five members formed the working group (steering committee) responsible for designing the guideline framework, consisting of 25 evidence-based recommendations.

Each recommendation was designed to address a specific clinical situation and assigned a recommendation grade following full panel voting. Three voting rounds were conducted using an online tool, with results statistically analysed by the steering committee. Following the first round of feedback, the base text was revised and rewritten. Second and third rounds of feedback were sought to refine the text, followed by adjustments to finalise the recommendation grades. Subsequently, the literature review was updated and organised to align with the evidence summaries supporting each recommendation. Finally, the manuscript was prepared for publication.

Three levels of evidence were considered: A—Data from more than one randomized clinical trial (RCT) or from meta-analyses of RCTs with low heterogeneity (I2 < 25%). B—Data from meta-analyses with high heterogeneity (I2 ≥ 25%), a single RCT, prespecified subgroup analysis, large observational studies, or meta-analyses of observational studies. C—Data from small or nonrandomized studies, exploratory analyses, clinical practice guidelines, or expert consensus statements. The level of agreement determined the strength of the recommendation, as follows: I—IS RECOMMENDED: > 90% agreement among panel members; IIa—SHOULD BE CONSIDERED: 70–90% agreement; IIb—MAY BE CONSIDERED: 50–70% agreement; and III—IS NOT RECOMMENDED: < 50% agreement or majority against. Recommendation grades and levels of evidence were established according to the guideline provided in Tables 1, 2.

**Table 1:**
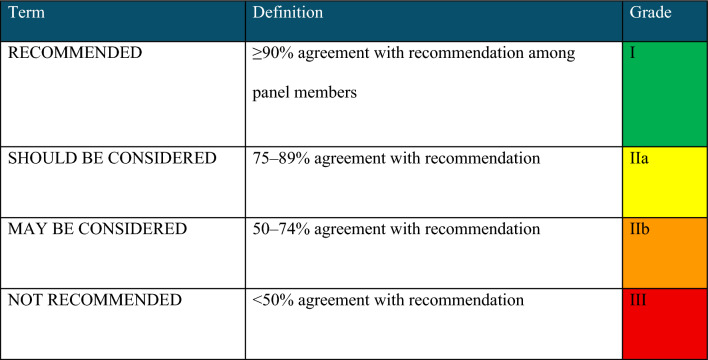
Recommendation grade

**Table 2:**
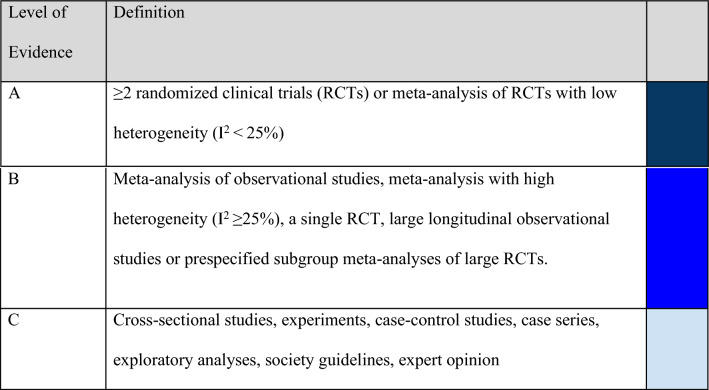
Level of evidence

## Results

### Part 1. Cardiovascular risk definition

#### Assessment of cardiovascular risk in individuals with overweight or obesity


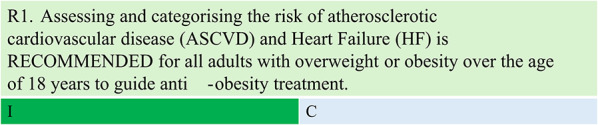


Summary of evidence (R1)

- Considering emerging evidence on the CV benefits of medications in reducing the risk of atherosclerotic CV disease (ASCVD) and HF in individuals with obesity, the selection of anti-obesity treatment should be guided by CV risk stratification. This recommendation is based on expert consensus.

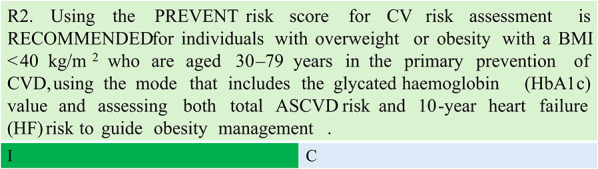


Summary of evidence (R2)

- The Predicting risk of CVD Events (PREVENT) equations was preferred over the older Pooled Cohort Equations due to its greater ethnic representativeness, larger population inclusion, and improved accuracy. However, the PREVENT score is limited in CVD subtype coverage. The PREVENT model incorporates expanded outcomes, including HF and risk factors related to obesity, diabetes, and kidney disease. The risk model demonstrates good prognostic performance with appropriate discrimination and calibration in both general populations and demographic/CV-kidney-metabolic subgroups. [15, 16]
- Designed for individuals with overweight or obesity, the PREVENT risk score should be applied using the mode that includes HbA1c measurement. Both total ASCVD risk and 10-year HF risk should be evaluated. The PREVENT score has limitations regarding age and BMI levels and should be restricted to patients aged 30 to 79 years with a BMI < 40 kg/m^2^. Additionally, the PREVENT score was developed for primary prevention patients – it should not be used for risk stratification in patients with established ASCVD and/or HF.

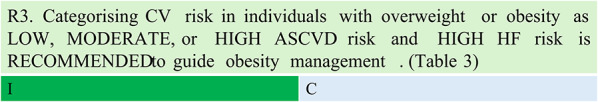


**Table 3:**
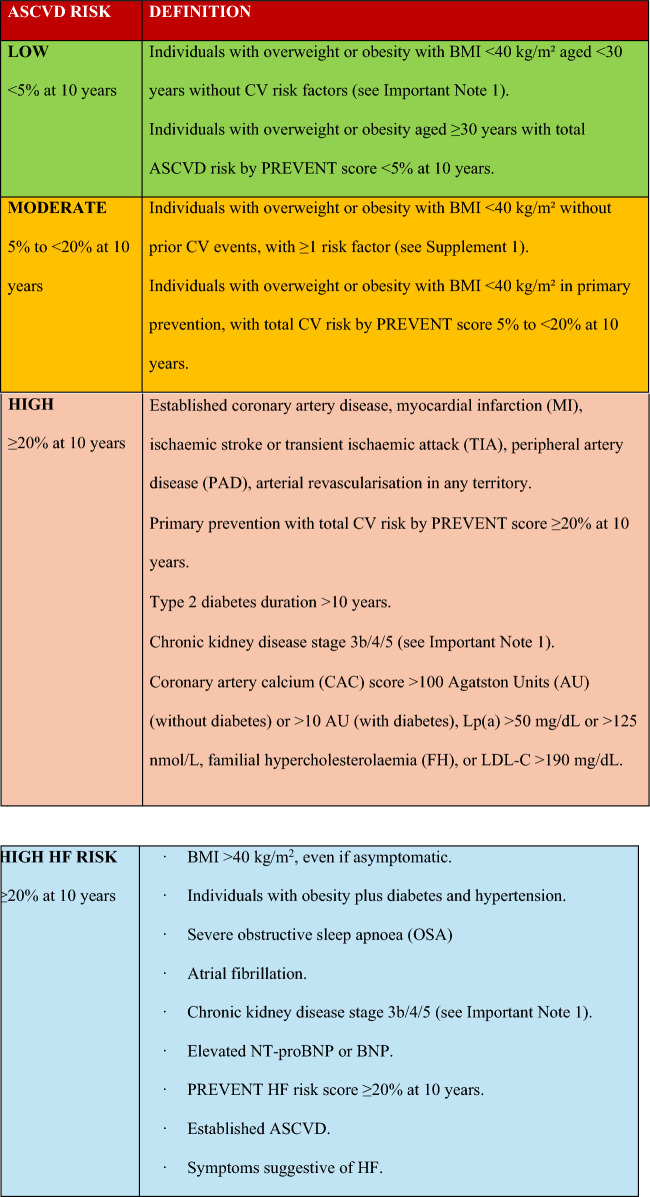
Cardiovascular risk assessment in individuals with overweight or obesity

Note**:** For convenience, the moderate risk category encompasses both borderline (5–7.5% over 10 years) and intermediate (7.5 to < 20% over 10 years) risk levels.

**Legends**

Agatston Units: AU; ASCVD: atherosclerotic cardiovascular disease; BMI: body mass index; CVD: Cardiovascular disease; HF: Heart failure; CAD: Coronary artery disease; CKD: Chronic kidney disease; CAC: Coronary artery calcium; FH: Familial hypercholesterolaemia; OSA: Obstructive sleep apnea; Lp(a): Lipoprotein(a); LDL-c: Low-density lipoprotein cholesterol; BNP: B-type natriuretic peptide, NT-proBNP: N-terminal pro-B-type natriuretic peptide

**Fig. 1:**
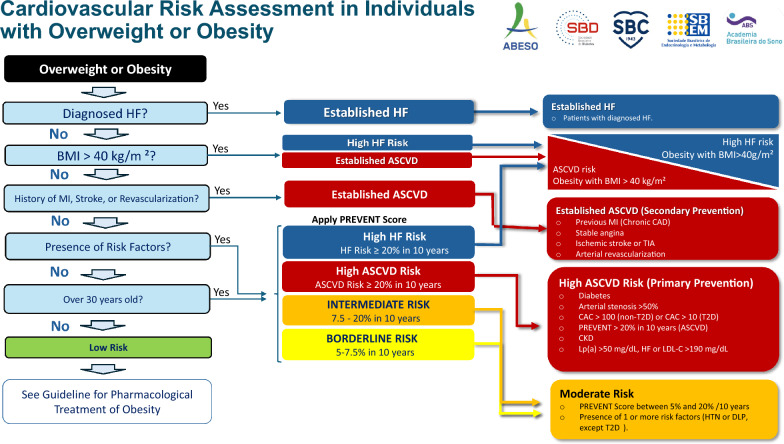
Cardiovascular risk assessment strategy in adults with overweight or obesity


The proposed strategy for CV risk assessment in adults with overweight or obesity is shown in Fig. 1.

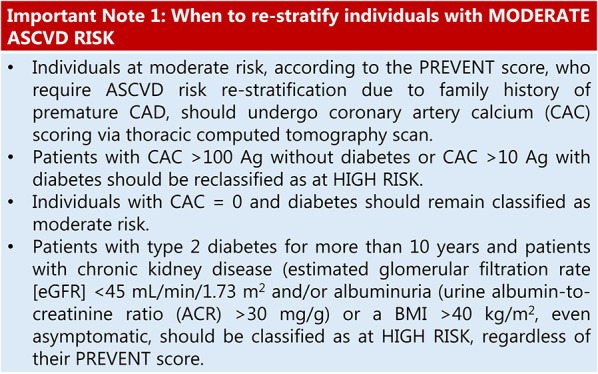


#### Heart failure screening in individuals with overweight or obesity


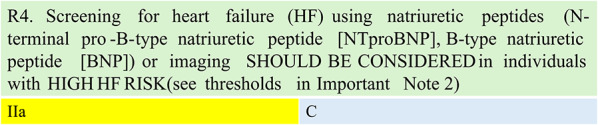


Summary of evidence (R4).

- Obesity is an important risk factor for HF with preserved ejection fraction (HFpEF). Individuals with obesity are often diagnosed late, by which time significant cardiac dysfunction may already be present. This delay is frequently due to the misattribution of symptoms, particularly in advanced stages, to obesity. [17]
- For individuals with obesity with HIGH HF risk, screening for type 2 diabetes, hypertension, atrial fibrillation, and obstructive sleep apnoea (OSA) and obtaining objective evidence of exercise intolerance can identify the need for HF-targeted interventions. Timely HF treatment improves prognosis regarding quality of life and morbidity/mortality. [18]

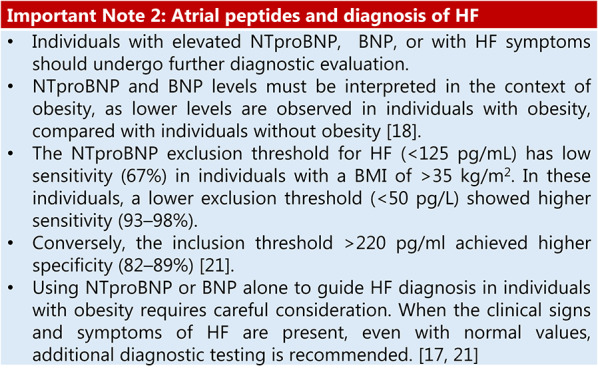

- The association between the apnoea-hypopnea index (AHI) and CV mortality is positive but of moderate magnitude. A meta-analysis found a hazard ratio [HR] of 2.07 (95% confidence interval [CI]: 1.48–2.91) for CV mortality in patients with severe OSA (AHI ≥ 30 events/hour) versus controls, while another study found a relative risk (RR) of 1.79 (95% CI: 1.47–2.18) for CV events at this severity level [19]. Dose-response analyses show that each 10-event/hour increase in AHI is associated with a 9–17% increase in CV event risk. The strength of this association varies by subgroup: the risk is more prominent in men under 70 years and individuals with excessive daytime sleepiness. The risk is lower or nonsignificant for mild-to-moderate OSA, indicating a dose-response effect with greater impact at higher AHI levels [20] (see Important Note 3).

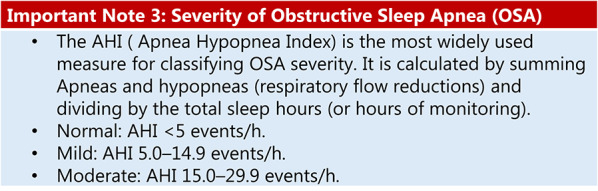


### Part 2: Weight loss targets

#### Weight loss targets for risk factor improvement


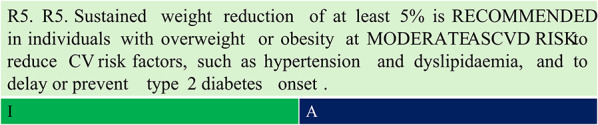


Summary of evidence (R5).

- Lifestyle modification (LSM) studies including the Diabetes Prevention Programme (DPP) and Look AHEAD study have demonstrated that modest weight loss of at least 5% of body weight significantly reduces cardiometabolic risk factors [22, 23].
- The DPP randomised 3,234 individuals with prediabetes or glucose intolerance to receive placebo, metformin (850 mg twice daily), or a lifestyle intervention targeting ≥ 7% weight loss and at least 150 min of physical activity per week. After 2.8 years of follow-up, lifestyle intervention reduced the incidence of diabetes by 58% (95% CI: 48–66%), while metformin reduced it by 31% (95% CI: 17–43%) compared with that observed with the placebo [22].
- The Look AHEAD study was a RCT that evaluated intensive lifestyle intervention versus diabetes support and education in 5,145 adults with overweight or obesity (mean BMI = 36 kg/m^2^) and type 2 diabetes. The primary endpoint was a composite of CV death, nonfatal myocardial infarction, nonfatal stroke, or hospitalisation for angina. The trial was terminated early due to futility after a median follow-up of 9.6 years. Although the primary endpoint was not met, intensive lifestyle intervention achieved higher weight loss (8.6% vs. 0.7% at 1 year; 6.0% vs. 3.5% at study end) and produced higher reductions in HbA1c and CV risk factors [23]. The magnitude of weight loss at 1 year was strongly associated with improvements in glycaemia, blood pressure, triglyceride levels, and high-density lipoprotein (HDL) cholesterol (p < 0.0001) but not with low-density lipoprotein (LDL) cholesterol (p = 0.79). Compared with stable-weight participants, individuals who lost 5 to < 10% (7.25 ± 2.1 kg) of body weight had greater odds of achieving HbA1c reduction (odds ratio [OR] 3.52 [95% CI: 2.81–4.40]), 5 mmHg reduction in diastolic blood pressure (OR 1.48 [1.20–1.82]), 5 mmHg reduction in systolic blood pressure (OR 1.56 [1.27–1.91]), 40 mg/dL reduction in triglyceride levels (OR 2.20 [1.71–2.83]), and 5 mg/dL increase in HDL cholesterol (OR 1.69 [1.37–2.07]) [24].

#### Weight loss targets for cardiovascular event reduction


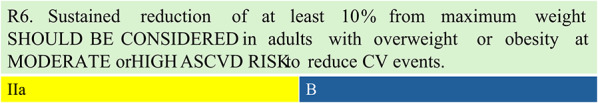


Summary of evidence (R6)

- The Da-Qing study was a Chinese RCT evaluating the effect of 6-year lifestyle intervention outcomes in 577 individuals with prediabetes and overweight (mean BMI = 25.7 kg/m^2^) on diabetes incidence, CV events, microvascular complications, CV death, all-cause mortality, and life expectancy. After 30 years of follow-up, reductions in CV events (HR 0.74, 95% CI: [0.59–0.92]), CV death (HR 0.67, 95% CI: [0.48–0.94], p = 0.022), and all-cause mortality (HR 0.74, 95% CI: [0.61–0.89], p = 0.0015) were observed, and diabetes diagnosis was delayed by up to 4 years [25].
- Although the primary endpoint in the Look-AHEAD study was not met, post hoc observational analysis suggests an association between initial weight loss magnitude and long-term CV event reduction in individuals with obesity and type 2 diabetes. Over a mean follow-up of 10.2 years (interquartile range [IQR] 9.5–10.7), individuals who lost ≥ 10% of body weight in the first year had 21% lower risk of the primary outcome (adjusted HR: 0.79, 95% CI: 0.64–0.98; p = 0.034) and 24% lower risk of the secondary outcome (adjusted HR 0.76, 95% CI: 0.63–0.91; p = 0.003) compared with that observed with those who had stable weight or weight gain. In the analyses using the control group as reference, participants who received intensive lifestyle intervention and lost ≥ 10% of body weight had 20% lower risk of the primary outcome (adjusted HR: 0.80, 95% CI: 0.65–0.99; p = 0.039) and 21% lower risk of the secondary outcome (adjusted HR 0.79, 95% CI: 0.66–0.95; p = 0.011) [26, 27].
- The Semaglutide Effects on Cardiovascular Outcomes in Individuals with Overweight or Obesity (SELECT) trial demonstrated the superiority of semaglutide 2.4 mg subcutaneous (SC) over placebo in reducing CV events in individuals with obesity and established CVD. Notably, the observed CV benefit was associated with a modest weight reduction of 9%.
- Subsequent analyses indicated that achieving much benefit is weight-loss independent, particularly for major adverse CV event (MACE) reductions observed early in the trial before significant weight loss was achieved. However, the contribution of weight loss to these benefits cannot be ruled out [28].
- Bariatric surgery has also shown reduction in CV events and mortality in populations with obesity. The Swedish Obese Subjects (SOS) study, a prospective, non-randomised cohort study, evaluated 4,047 individuals with obesity (BMI ≥ 34 kg/m^2^ for men and ≥ 38 kg/m^2^ for women), of whom 2,010 underwent bariatric surgery (gastric banding, vertical banded gastroplasty, or gastric bypass) and 2,037 received conventional obesity treatment. The mean follow-up duration was 14.7 years. Mean weight loss in the surgical group at 10 years was 17% (vs. 1% in the conventional treatment group). For composite CV events (fatal and nonfatal myocardial infarction, fatal and nonfatal stroke, angina pectoris, and HF), the surgical group showed a significant 33% reduction, plus 53% reduction in CVD death (HR 0.47; 95% CI: 0.29–0.76; p = 0.002) compared with that observed with conventional treatment. Although surgery-specific effects cannot be excluded, the fact that > 80% of the procedures were restrictive (vertical banded gastroplasty and gastric banding, where hormonal effects contributing to weight loss are less relevant) indicates that the main factor associated with MACE reduction was significant (> 15%), and it sustained weight loss [29].
- Based on the available evidence, the panel recommends a weight loss target of at least 10% from maximum lifetime weight [30] for individuals at moderate or high ASCVD risk, as a strategy to reduce CV events.

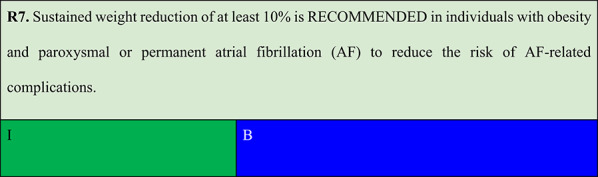


#### Weight loss targets for atrial fibrillation- related complications

Summary of evidence (R7):

- Weight reduction in individuals with obesity has demonstrated a positive impact on reducing symptom burden and AF recurrence [31].
- A single-centre, partially blinded RCT conducted in Australia that enrolled 150 individuals with obesity or overweight and AF showed that a structured weight management programme significantly reduced symptom burden, severity, and number of AF episodes over 15 months of follow-up. The intervention group compared with the control group lost more weight (14.3 kg vs. 3.6 kg) and had higher reductions in symptom severity scores and interventricular septal thickness [32].
- Another meta-analysis revealed that weight loss of at least 10% is associated with lower AF recurrence, reduced AF burden, and improved symptom severity [33]. Similarly, weight loss following catheter ablation reduced AF recurrence at 12 months of follow-up [34].
- In the SOS study, bariatric surgery reduced the risk of new-onset AF compared with that observed with usual care. Risk reduction was more pronounced in younger individuals and those with elevated diastolic blood pressure [35].

### Part 3. Obesity management

#### Lifestyle modification


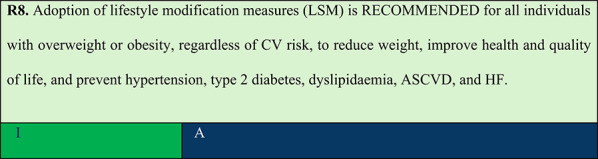


Summary of evidence (R8):

- Lifestyle modification measures (LSM) in individuals with overweight or obesity should include a dietary programme with appropriate and healthy macronutrient distribution combined with aerobic and resistance exercises [36].
- The LSM approach should be multidisciplinary, with a team including a dietitian, exercise physiologist and psychologist, delivered through individual or group sessions. Incorporation of LSM should not delay the initiation of anti-obesity pharmacotherapy, when indicated [37].
- Nutritional counselling should focus on reducing portion sizes, increasing intake of fruits and vegetables, and reducing consumption of alcohol and ultra-processed foods. Moreover, it should target an initial energy deficit of 500–750 kcal/day, which will need further adjustments based on body weight and individual activity levels [37].

- RCTs evaluating medications with moderate weight loss effects, including sibutramine, [38], liraglutide [39], and bupropion/naltrexone combination [40], demonstrated that combination with LSM produced superior results for body weight reduction and cardiometabolic risk factors.
- The advent of more potent anti-obesity medications, such as semaglutide [41] and tirzepatide [42], has led to the possibility of greater caloric deficits and, as a result, enhanced weight reduction. In this context, it is essential to closely monitor the consumption of various macronutrients, particularly protein, to avoid sarcopenia and nutritional deficiencies. This approach will ensure the establishment of healthy and sustainable eating habits [43].

- Similarly, a healthy diet can be achieved with dietary patterns rich in fresh and minimally processed foods, such as Mediterranean and Dietary Approaches to Stop Hypertension diets, which include whole grains, fruits and vegetables, lean white meats, and plant protein sources such as legumes and nuts [44, 45]. These dietary patterns reduce cardiometabolic risk [45] and may serve as references but should be adapted for Brazilian preferences and contexts to support adherence [46]. Limiting the intake of ultra-processed foods rich in saturated fats and refined sugars is also recommended, as these have been linked to poorer body composition and elevated all-cause and cardiovascular mortality rates [47].

## Pharmacotherapy management for reducing events and risk factors

### Obesity and MODERATE or high ASCVD risk


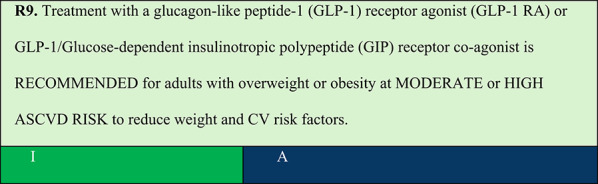


Summary of evidence (R9):

- Liraglutide, a GLP-1 RA with weight loss efficacy at 3.0 mg/day, had its effects on obesity and complications evaluated in the Satiety and Clinical Adiposity–Liraglutide Evidence (SCALE) programme. The SCALE Obesity and Prediabetes trial randomised 2,254 patients to receive liraglutide 3.0 mg or placebo. After 56 weeks, 63.2% and 33.1% of the patients lost > 5% and > 10% of initial weight, respectively. After 3 years, the risk of developing diabetes was reduced by 79% in patients with prediabetes; patients on liraglutide compared with those on placebo took 2.7 times longer to develop diabetes [48].
- A post hoc analysis using pooled data from 5,908 individuals across 5 RCTs in the SCALE programme (liraglutide vs. placebo or orlistat) demonstrated the CV safety of liraglutide 3.0 mg in individuals with obesity [49].
- The Semaglutide Treatment Effect in Individuals with Obesity (STEP 1) trial included 1,961 patients with overweight or obesity without type 2 diabetes who were followed up for 68 weeks. All individuals received a reduced-calorie diet with 500 kcal/day deficit and counselling for 150 min of weekly physical activity. At the end of the study, participants in the semaglutide 2.4 mg group lost 16.9% of body weight, with nadir around week 60, while the placebo group lost 2.4% [50].
- Tirzepatide, a GLP-1/GIP receptor co-agonist, also demonstrated efficacy in reducing progression to diabetes in patients with obesity and prediabetes. In an analysis of SURMOUNT-1 including 1,032 individuals with obesity and prediabetes treated with tirzepatide for approximately 3 years (176 weeks), type 2 diabetes incidence was lower than that observed with the placebo (1.3% vs. 13.3%; HR 0.07 [95% CI: 0.0–0.1; p < 0.001). Additionally, after 17 weeks of tirzepatide discontinuation, 2.4% of the tirzepatide group vs. 13.7% of the placebo group developed type 2 diabetes (HR 0.12, 95% CI: 0.1–0.2; p < 0.001). In absolute terms, 99% of individuals with prediabetes who received tirzepatide remained diabetes-free. Mean weight loss in the tirzepatide 5 mg, 10 mg, and 15 mg groups was −12.3%, −18.7%, and −19.7%, respectively, versus −1.3% in the placebo group at 3 years (p < 0.001 versus placebo for all comparisons). Furthermore, weight loss exceeding 20% was associated with an HR for type 2 diabetes progression of 0.07, with a number need to treat (NNT) of 9 to prevent one case, and with 92% of patients achieving normoglycaemia, reinforcing the importance of weight loss in diabetes prevention [51].
- Regarding individuals with obesity and diabetes, a systematic review evaluated the effect of non-insulin antidiabetic medications on weight loss in individuals with type 2 diabetes across multiple RCTs. Liraglutide, semaglutide, and tirzepatide resulted in greater weight loss compared with that observed with other therapeutic classes (weight loss > 5%) [52].

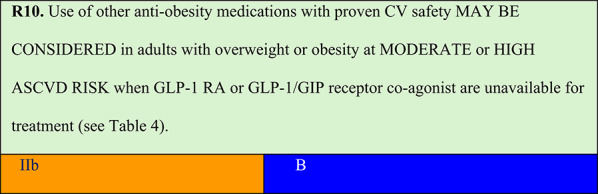


Summary of evidence (R10):

- Orlistat is a gastric and pancreatic lipase inhibitor that promotes weight reduction by reducing the absorption of 30% of ingested fat. The ‘Xenical in the prevention of diabetes in obese subjects’ study randomised 3,305 individuals with obesity (BMI ≥ 30 kg/m^2^) and normal glycaemia (79%) or impaired glucose tolerance (21%) to orlistat (120 mg TID) or placebo, both with LSM. After 4 years of treatment, cumulative diabetes incidence was 9% in the placebo group vs. 6.2% in the orlistat group, corresponding to a risk reduction of 37.3% (p = 0.0032). Exploratory analysis demonstrated that greater weight loss was the primary determinant of diabetes prevention. Over 4 years, individuals using orlistat lost more weight than that observed using placebo (5.8 versus 3.0 kg, respectively; p < 0.001) [53].
- In a meta-analysis of four RCTs evaluating naltrexone/bupropion combination versus placebo at 1 year, the weight loss difference was 5.0 kg (95% CI: 3.96–5.94). Compared with placebo, 55% (48–61%) of patients taking the medication achieved ≥ 5% weight loss, and 30% (24–37%) achieved ≥ 10% weight loss [54]. In the Contrave Obesity Research-Diabetes study evaluating patients with type 2 diabetes, treated patients had a mean reduction of 11.2% in triglycerides (versus −0.8% with placebo) and an increase of 3.0 ± 0.5 mg/dL in HDL cholesterol (versus −0.3 ± 0.6 mg/dL with placebo), with no significant effect on LDL cholesterol [55].
- CV safety of naltrexone/bupropion was evaluated in the LIGHT trial (n = 4,454), which was terminated early following the public disclosure of confidential interim data. However, 50% of the pre-specified events had occurred. MACEs were reported in 102 patients (2.3%) in the placebo group and 90 patients (2.0%) in the naltrexone/bupropion group (HR 0.88; 99.7% CI 0.57–1.34). These findings support the CV safety of naltrexone/bupropion over a mean follow-up period of 2 years, during which weight loss was maintained [56]. A subsequent systematic review and meta-analysis further confirmed the CV safety profile of the therapy [57].
- Medications approved in Brazil for obesity treatment that have presented superior weight loss and CV safety when compared to placebo are listed in Table 4. The most common adverse events and contraindications observed are listed in Table 5 [58].

**Table 4:**
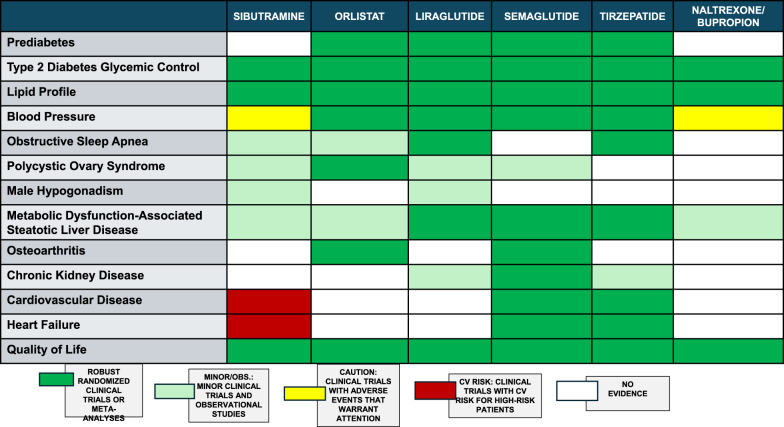
Summary of the main effects of approved anti-obesity medications in Brazil

*Studies primarily designed for diabetes treatment were excluded.

**Table 5:** Most common and specific side effects of the anti-obesity pharmacologic agents

Medication	More than 10% of patients	Specific side effects that deserve attention
Sibutramine 10–15 mg	Constipation, xerostomia, insomnia	Tachycardia/increased heart rate, increased blood pressure, headache, anxiety
Orlistat 120 mg 3 x/day	Diarrhea/steatorrhea/urgency, flatulence, upper respiratory tract infections/flu, headache, hypoglycemia	Hypersensitivity reactions, long-term deficiency of fat-soluble vitamins
Liraglutide 3.0 mg/day	Nausea and vomiting, diarrhea, constipation	Injection site reactions, increased heart rate, insomnia, cholelithiasis, asthenia and fatigue, hypoglycemia
Semaglutide 2.4 mg/week	Nausea and vomiting, diarrhea, constipation, abdominal pain, headache, fatigue	Injection site reactions, increased heart rate, cholelithiasis, hypoglycemia
Tirzepatide 10 and 15 mg/week	Hypoglycemia (when used with sulfonylureas or insulin), nausea, diarrhea	Hypersensitivity reactions, increased heart rate, injection site reactions
Naltrexone/Bupropion 360/32 mg/day	Nausea, constipation, headache, vomiting	Suicidal thoughts or actions, seizures, risk of opioid overdose, sudden opioid withdrawal, severe allergic reactions, increased blood pressure or heart rate, hepatitis, manic episodes, narrow-angle glaucoma, hypoglycemia (when used with sulfonylureas or insulin), serotonin syndrome

### Obesity and established ASCVD risk


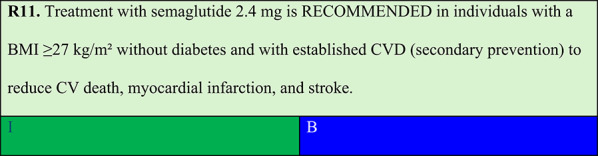


Summary of evidence (R11):

- The SELECT trial was an RCT including 17,604 individuals, with overweight or obesity with a mean age of 61.6 years and BMI of 33.34 kg/m^2^, designed to evaluate the secondary prevention of CV events. The study population had no prior diabetes diagnosis, with established CV disease as an inclusion criterion. Mean weight loss of 9% was achieved. The results demonstrated a 20% reduction in MACE (CV death, nonfatal myocardial infarction, and nonfatal stroke) (6.5% versus 8.0% with placebo, HR 0.80, 95% CI: 0.72–0.90, p < 0.001) [59].

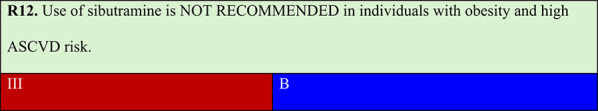


Summary of evidence (R12):

- The *‘*Effect of Sibutramine on Cardiovascular Outcomes in Overweight and Obese Subjects’ (SCOUT) study evaluated sibutramine versus placebo in individuals with overweight/obesity, prior CVD and/or type 2 diabetes plus one CV risk factor. The risk of a primary endpoint event (nonfatal myocardial infarction, nonfatal stroke, resuscitation after cardiac arrest, or CV death) was 11.4% in the sibutramine group compared with 10.0% in the placebo group (HR 1.16; 95% CI: 1.03–1.31; p = 0.02). Therefore, this panel considers sibutramine use not recommended in individuals with obesity and high ASCVD risk or chronic CAD [60].

### Obesity and type 2 diabetes


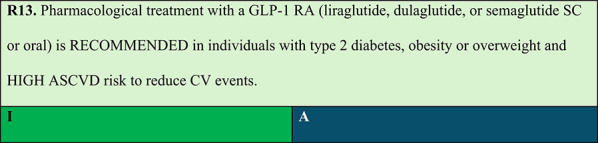


Summary of evidence (R13):

- Six CV outcome RCTs—LEADER, SUSTAIN-6, REWIND, HARMONY, AMPLITUDE-O, and SOUL—consistently demonstrated the efficacy and safety of GLP-1 RAs in individuals with type 2 diabetes, additionally showing secondary protective effects in individuals with type 2 diabetes and CVD [61].
- A systematic review with meta-analysis of RCTs in patients with type 2 diabetes demonstrated that GLP-1 RAs significantly reduce composite MACE (CV death, nonfatal myocardial infarction, and nonfatal stroke) by 14% and all-cause mortality by 12%. GLP-1 RAs also reduced HF hospitalisations by 11% and composite kidney outcomes by 21%. Notably, these clinical benefits occur without increased risk of severe hypoglycaemia, retinopathy, or pancreatic adverse events, reinforcing the safety profile of these agents in managing obesity and type 2 diabetes [62].
- A systematic review with network meta-analysis demonstrated that GLP-1 RAs significantly reduced all-cause and CV mortality, as well as incidence of nonfatal myocardial infarction, nonfatal stroke, kidney failure, and HF hospitalisations in individuals with type 2 diabetes [63].
- The SOUL trial is a double-blind, placebo-controlled RCT that evaluated the CV efficacy of oral semaglutide in 9,650 patients with type 2 diabetes and ASCVD, chronic kidney disease (CKD), or both. After a mean follow-up of 47.5 months, oral semaglutide significantly reduced MACE risk, including CV death, nonfatal myocardial infarction, and nonfatal stroke. Event incidence was 3.1 per 100 person-years in the semaglutide group versus 3.7 per 100 person-years in the placebo group, yielding a 14% relative risk reduction (HR 0.86; 95% CI: 0.77–0.96; p = 0.006) [64].

### Obesity, type 2 diabetes, and chronic kidney disease


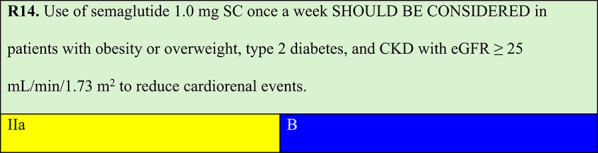


Summary of evidence (R14):

- The ‘Effect of semaglutide versus placebo on the progression of renal impairment in individuals with type 2 diabetes and chronic kidney disease’ (FLOW) trial was a multicentre study that included 3,534 participants with type 2 diabetes, CKD, and overweight or obesity to investigate the effect of weekly SC semaglutide on kidney disease progression. The composite primary endpoint was persistent eGFR decline of ≥ 50% from baseline, end-stage kidney disease, death from kidney disease, or CV death. The trial was stopped early for efficacy. The trial achieved a significant 24% reduction in kidney disease progression and CV and kidney mortality for individuals treated with semaglutide 1.0 mg. Additionally, semaglutide 1.0 mg had positive impacts on other clinical outcomes: 21% reduction in CV death risk (HR 0.71; 95% CI: 0.56–0.89), 21% reduction in composite renal outcomes (HR 0.79; 95% CI: 0.66–0.94), 18% reduction in the risk of severe CV events (HR 0.82; 95% CI: 0.68–0.98; p = 0.029), and a 20% reduction in all-cause mortality (HR 0.80; 95% CI: 0,67–0,95; p = 0,01) [65].

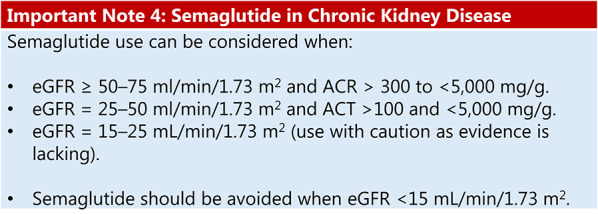


### Obesity and obstructive sleep apnoea


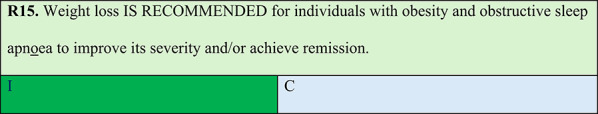


Summary of evidence (R15):

- Evidence regarding the impact of obesity treatment on obstructive sleep apnoea severity and remission has gained increasing attention but still presents significant limitations [66].
- Regarding non-pharmacological measures, small, randomised studies with short follow-up durations have indicated that interdisciplinary weight reduction strategies can reduce obstructive sleep apnoea severity, particularly in milder cases [67, 68].
- Remission (normalisation of the apnoea-hypopnea index without need for specific treatments, such as continuous positive airway pressure (CPAP)), may occur in some cases. Importantly, to achieve these results, some studies have adopted highly restrictive intervention measures that are difficult to implement broadly in the long-term [69]

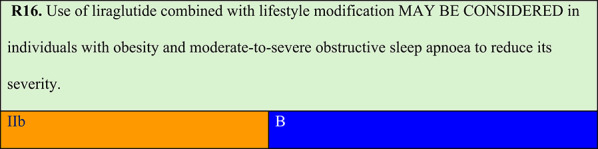


Summary of evidence (R16):

- Regarding pharmacological obesity treatment, randomised trial evidence is currently limited to two studies that have tested two medications (liraglutide and tirzepatide) [70, 71].
- The SCALE study tested liraglutide 3.0 mg daily for 32 weeks in individuals with obesity without diabetes who had moderate or severe OSA and were not using or had not tolerated CPAP adjunctive to diet and exercise. After 32 weeks, mean AHI reduction was greater with liraglutide than with placebo (−12.2 vs. −6.1 events/h), paralleling greater mean weight loss with liraglutide versus placebo (−5.7% vs. −1.6%). This study showed that residual AHI remained significant, suggesting that patients did not achieve OSA remission [70].

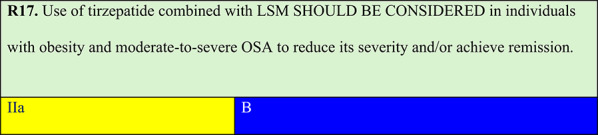


Summary of evidence (R17):

- The SURMOUNT-OSA trial was a multicentre RCT including 469 patients with obesity and moderate-to-severe OSA, with or without prior CPAP use, randomised to the tirzepatide or placebo group. Compared with the placebo, tirzepatide treatment at 10–15 mg weekly for 52 weeks resulted in 16–17% weight reduction in both sub studies (with or without prior CPAP use). Weight loss was accompanied by AHI reductions of 20 and 24 events per hour compared with that observed with the placebo, and relative event reductions of 48% and 56% in patients with and without CPAP, respectively. A significant proportion achieved OSA remission or ‘non-clinically relevant’ apnoea (mild or asymptomatic) [71].

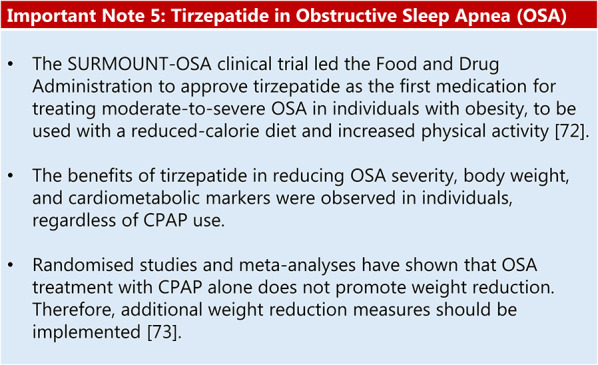


### Obesity and heart failure


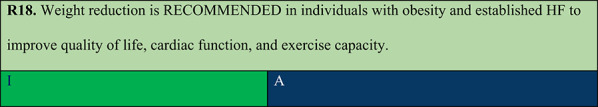


Summary of evidence (R18):

- A meta-analysis of 19 RCTs and observational studies involving 449,882 individuals with obesity showed that weight loss, although it does not reduce mortality, improves quality of life, ventricular function, and exercise capacity [74].
- A meta-analysis of 29 studies showed that intentional weight loss through interventions, such as bariatric surgery, can improve cardiac function and quality of life in patients with HF and obesity. Bariatric surgery was associated with reduced risk of developing HF and improvements in diastolic function and left ventricular mass. A J-curve was observed between BMI and HF risk, with maximum risk in severe obesity (BMI > 40 kg/m^2^) of 1.73 (95% CI: 1.30–2.31), p < 0.001). Although the obesity paradox was observed for all-cause mortality, the overweight group was associated with lower CV mortality (OR 0.86, 95% CI: 0.79–0.94), with no significant difference among other BMI categories. Bariatric surgery-induced weight loss in individuals with obesity without established HF, atrial fibrillation, or known CAD was associated with reduced left ventricular mass (p < 0.0001), improved left ventricular diastolic function (p ≤ 0.0001), and reduced left atrial size (p = 0.02) [75].

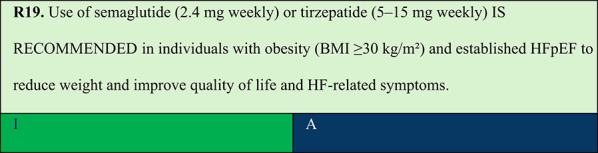


Summary of evidence (R19):

- In individuals with established HFpEF, three clinical trials have demonstrated efficacy in improving HF-related outcomes.
- Two RCTs evaluated semaglutide 2.4 mg once weekly in individuals with HFpEF and obesity, demonstrating that the GLP-1 agonist improved HF-related symptoms, functional capacity, and body weight [76, 77].
- The STEP-HFpEF trial was an RCT comparing semaglutide SC 2.4 mg versus placebo in 529 individuals with obesity, NYHA class II–IV HF, elevated natriuretic peptide levels (with BMI-stratified thresholds at the start of the study), left ventricular ejection fraction > 45%, and evidence of echocardiographic abnormalities. Most participants (84%) had left ventricular ejection fraction ≥ 50%. Treatment with semaglutide 2.4 mg once weekly for 1 year resulted in a significant reduction in body weight (13.3% vs. 2.6% with placebo) and improvements in the Kansas City Cardiomyopathy Questionnaire Clinical Summary Score (KCCQ-CSS) and 6-min walk distance. Additionally, the reduction in NT-proBNP levels was approximately 15% greater with semaglutide than with the placebo [76].
- The STEP-HFpEF DM trial compared semaglutide 2.4 mg SC with placebo in individuals with obesity and type 2 diabetes. The results paralleled that of the STEP-HFpEF: semaglutide led to greater reductions in HF-related symptoms and physical limitations and higher weight loss after 1 year of treatment [77].
- The SUMMIT trial was a 104-week RCT evaluating tirzepatide (titrated to 15 mg SC weekly; n = 364) versus placebo (n = 367) in patients with NYHA class II–IV HF, ejection fraction ≥ 50%, and BMI ≥ 30 kg/m^2^. Worsening HF events occurred in 29 patients in the tirzepatide group (8.0%) and 52 patients in the placebo group (14.2%) (HR 0.54; 95% CI: 0.34–0.85). CV death occurred in 8 patients (2.2%) and 5 patients (1.4%), respectively (HR 1.58; 95% CI: 0.52–4.83). Treatment with tirzepatide, compared with placebo, reduced the composite endpoint of CV or HF worsening and improved multiple health status measures in this population, including the KCCQ-CSS, 6-min walk distance, health status index, and Patient Global Impression of Severity Overall Health Score [78].

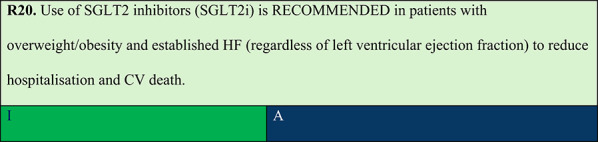


Summary of evidence (R20):

- A pre-specified meta-analysis of the DELIVER and EMPEROR-Preserved trials (n = 12,251) demonstrated that SGLT2i significantly reduced the risk of CV death or HF hospitalisation in individuals with preserved or mildly reduced ejection fraction (HR 0.80; 95% CI: 0.73–0.87) [79]. In the DAPA-HF, EMPEROR-Reduced (reduced ejection fraction), and SOLOIST-WHF (varied ejection fraction) trials, the analysis of 21,947 individuals confirmed reductions in CV death or HF hospitalisation (HR 0.77 [95% CI: 0.72–0.82]); CV death (HR 0.87 [95% CI: 0.79–0.95]); first HF hospitalisation (HR 0.72 [95% CI: 0.67–0.78]); and all-cause mortality (HR 0.92 [95% CI: 0.86–0.99]). The benefits were consistent across all subgroups, including different ejection fraction ranges.

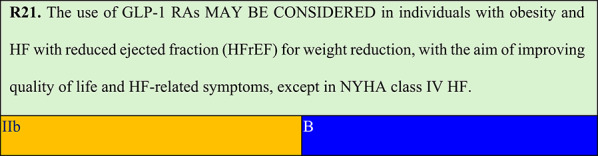


Summary of evidence (R21):

- In patients with HFrEF, evidence for obesity treatment with GLP-1 RAs is insufficient, and their safety remains debated.
- In the FIGHT study, individuals with recent HFrEF hospitalisation (mean ejection fraction, 27%) randomised to receive liraglutide showed a numerical but nonsignificant increase in HF hospitalisations [80].
- A post hoc analysis of the same study among individuals who received liraglutide for at least one follow-up visit showed significant and safe weight reduction in this population (−1.96 kg, approximately −4.1 pounds). The population had a median age of 61 years, 21% were female, 69% had NYHA class III or IV, and the median ejection fraction was 25% (IQR 19–32%) [81].
- In the LIVE study including individuals with chronic HFrEF allocated to the liraglutide group, there was also an increased risk of adverse cardiac events, although only one death and one HF hospitalisation occurred. Notably, the total number of adverse cardiac events was low (12 [10%] with liraglutide vs. 3 [3%] with placebo, p = 0.04) [82].
- Importantly, neither study (FIGHT or LIVE) aimed to treat HFrEF in individuals with obesity, and liraglutide was not used at obesity treatment doses.
- In a post hoc analysis of SELECT including 1,347 individuals with HFrEF (mean BMI 33.4 kg/m^2^), semaglutide reduced MACE risk by 35% and the composite of CV death and HF hospitalisation/urgent visit by 21%, although the effect on HF hospitalisations alone was not significant (HR 1.08; p = 0.11). Notably, approximately 60% of included individuals had NYHA class II, and individuals with NYHA class IV HF were excluded. Additionally, the adverse event rate during follow-up was low [83].
- In a pre-specified analysis of STEP-HFpEF and STEP-HFpEF DM, semaglutide effects on primary outcomes and body weight were similar across three groups based on baseline ejection fraction (45–49%, 50–59%, and ≥ 60%). Similarly, left ventricular ejection fraction did not influence semaglutide results for the following confirmatory secondary endpoints: 6-min walk distance (interaction p = 0.19), hierarchical composite endpoint (interaction p = 0.43), and high-sensitivity C-reactive protein [84]. Despite these results, all GLP-1 RAs, including semaglutide, are associated with a modest increase in heart rate (3–5 bpm) [85].

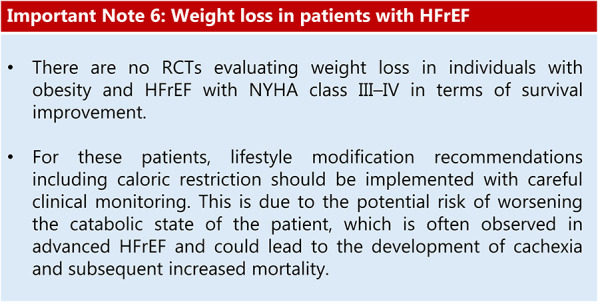


### Obesity in individuals with high heart failure risk


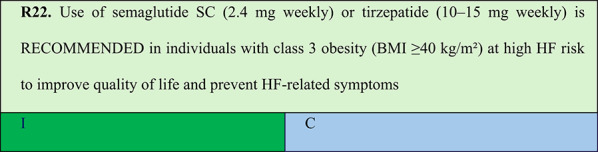


Summary of evidence (R22):

- Based on expert opinion, this panel recommends weekly use of either semaglutide 2.4 mg SC or tirzepatide 10–15 mg for potential prevention of HF-related outcomes in patients with class 3 obesity or at high HF risk. The panel based its recommendation on studies conducted in patients with established HF [76, 78], as well as on the plausible benefits in this population, given that HF represents a continuous pathophysiological progress through interconnected stages driven by various risk factors, among which obesity plays a central role [17].

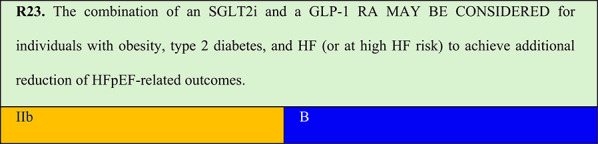


Summary of evidence (R23):

- Real-world studies, meta-analyses of RCTs, observational studies, and retrospective studies have indicated a potential additive effect of SGLT2i/GLP-1 RA combination over monotherapy with either agent. This effect, however, requires confirmation in RCTs. This panel assessed by expert opinion that there is plausibility for additive effects of this combination, as these agents act through different mechanisms, with potential for additive benefit. This panel indicates that the SGLT2i/GLP-1 RA combination should be considered for patients with obesity and HFpEF or at HF risk as it has been associated with greater improvement of HFpEF-related outcomes when compared with monotherapy alone.
- A systematic review with meta-analysis indicates that cardiorenal benefits may be enhanced with combination therapy compared with monotherapy. The study evaluated the cardiorenal effects of combining SGLT2i with GLP-1 RA compared with monotherapy with each agent class in patients with type 2 diabetes. Eligible studies were RCTs and observational studies comparing SGLT2i or GLP-1 RA in combination or as a monotherapy. Five RCTs and 10 post hoc observational analyses were identified. Compared with GLP-1 RA monotherapy, combination therapy with SGLT2i and GLP-1 RA was associated with lower risk of HF-related outcomes (RR 0.63, 95% CI: 0.51–0.77, p < 0.001) and all-cause mortality (RR 0.66, 95% CI: 0.50–0.88, p = 0.004) in patients with type 2 diabetes [86].
- A retrospective real-world study from a Spanish database included 15,549 individuals with type 2 diabetes from 2018 to 2022, with 46% having obesity, 71% having hypertension, 15% having CAD, and 10% established HF. Three groups were established according to the therapy used: 1) SGLT2i monotherapy (n = 12,029; mean duration: 14 months), 2) GLP-1 RA monotherapy (n = 1,071; mean duration: 17 months), or 3) GLP-1 RA + SGLT2i (n = 2,449; mean duration: 14 months). Data were analysed using 1:1 propensity score matching. The median follow-up duration was 19 (8–33) months. Combination therapy versus SGLT2i reduced the risk of HF events (HR 0.69; 95% CI: 0.56–0.87) and all-cause mortality (HR 0.68; 95% CI: 0.54–0.86). Multivariate Cox regression after propensity score matching confirmed the benefit of combination therapy compared with SGLT2i and GLP-1 RA monotherapy. Combined SGLT2i and GLP-1 RA therapy was associated with reduced risk of HF events and all-cause mortality compared with that observed with monotherapy in this population [87].

## Bariatric surgery

### Obesity stage 2 and moderate/high ASCVD risk or high hf risk


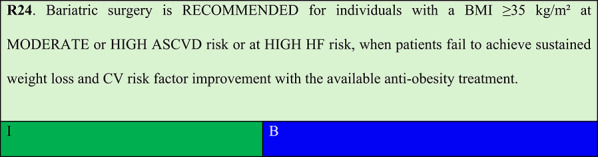


Summary of evidence (R24):

- A systematic review and meta-analysis of 18 observational studies from population databases including over 1.5 million patients evaluated obesity-related disease incidence and overall mortality after a minimum 18-month follow-up in bariatric surgery versus control groups. The analysis identified that bariatric surgery is associated with reduced all-cause mortality (OR 0.62; 95% CI: 0.55–0.69; p < 0.001) and CV mortality (OR 0.50; 95% CI: 0.35–0.71; p < 0.001). Additionally, there was reduced incidence of type 2 diabetes (OR 0.39; 95% CI: 0.18–0.83), hypertension (OR 0.36; 95% CI: 0.32–0.40), and dyslipidaemia (OR 0.33; 95% CI: 0.14–0.80) [89].
- A longitudinal cohort study evaluated 1,724 patients undergoing bariatric surgery (gastric banding and Roux-en-Y gastric bypass), compared with controls matched for age, BMI, sex, and Framingham score receiving conventional medical treatment and followed up for up to 12 years (median 6.3 years). Surgery was associated with a 42% reduction in MACE risk (HR 0.58; 95% CI: 0.42–0.82; p = 0.0018), including myocardial infarction, stroke, and congestive HF. The reduction in congestive HF was particularly marked (HR 0.38; 95% CI: 0.22–0.64; p = 0.0003). Improvements in CV risk factors (total cholesterol, HDL cholesterol, and blood pressure) occurred within 1 year and Framingham score improvements within 2 years [90].
- Concurrently, an observational study evaluated 20,235 individuals with class 2 or 3 obesity and type 2 diabetes from 2005 to 2010 in the United States, where 5,301 underwent bariatric surgery and 14,934 served as controls, matched for age, sex, BMI, and HbA1c. The primary outcome was incidence of acute myocardial infarction, unstable angina, percutaneous coronary intervention, or coronary artery bypass grafting. After 5 years of follow-up, the bariatric surgery group had lower incidence of the primary outcome compared with the non-surgical group: 2.1% vs. 4.3% (HR 0.60, 95% CI: 0.42–0.86), respectively. There was also lower CAD incidence in the surgical group compared with the non-surgical control group: 1.6% vs. 2.8% (HR 0.64, 95% CI: 0.42–0.99) [91].
- The SOS study demonstrated that bariatric surgery is associated with reduced risk of developing HF in individuals with severe obesity versus those with obesity under usual care [92].
- In these studies, the effects of bariatric surgery on CV event reduction were observed progressively following normalisation of metabolic parameters including blood pressure, lipid profile, and glycaemic control. MACE reduction cannot be attributed to direct or immediate surgical effects on the CV system, as CV risk factor improvements occurred after sustained weight loss, and comparisons were made with less effective anti-obesity pharmacotherapy treatment. This panel considers that, in the absence of specific RCTs, the indication of bariatric surgery for CV event prevention should be considered based on its long-term benefits in improving risk factors, particularly in patients at moderate or high CV risk who either lack access to or do not achieve a sustained response with currently available anti-obesity therapies.

### Obesity stage 2 and heart failure


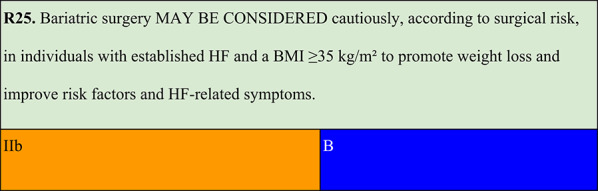


Summary of evidence (R25):

- Although severe HF or marked systolic dysfunction may be considered as a contraindication to bariatric surgery, emerging evidence has indicated that the procedure may be appropriate for select patients with obesity and stable HF.
- A systematic review with meta-analysis demonstrated that bariatric surgery is associated with reduced HF-related hospitalisations, as well as improvements in left ventricular ejection fraction and NYHA functional class [93].
- Bariatric surgery has also shown benefits in reducing CV risk factors and improving cardiac function, including reversal of cardiac remodelling and improvements in both systolic and diastolic performance [94].

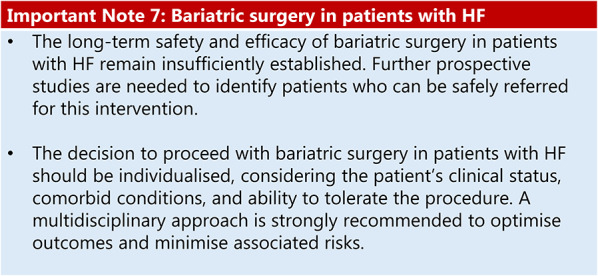


**Fig. 2:**
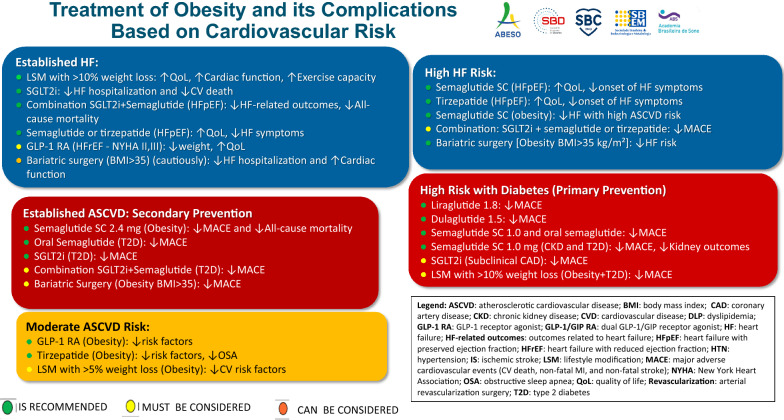
Treatment of obesity and its complications based on cardiovascular risk categories


As shown in Fig. 2, the flowchart provides a summary of the management of obesity and its complications, guided by cardiovascular risk assessment.

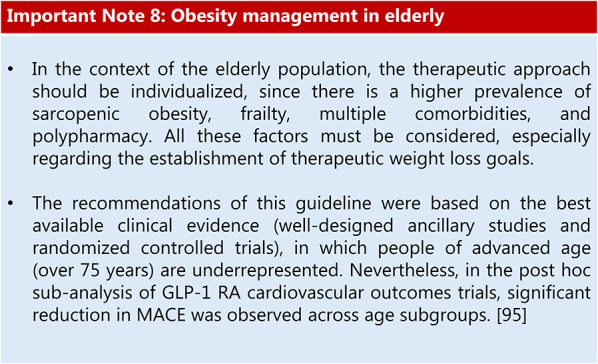



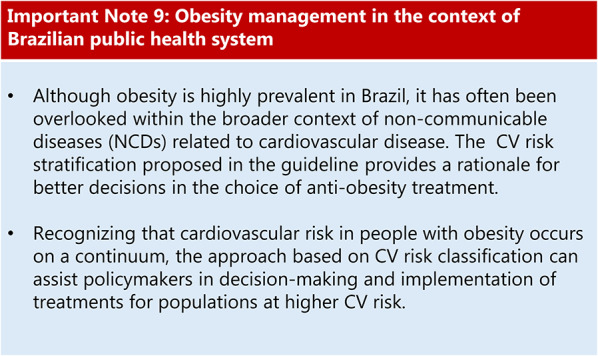


## Conclusion

Considering the increasing incidence of obesity and its well-established association with CVD and related outcomes, CV risk assessment must be a central component in obesity treatment planning. This guideline, developed through a collaboration among five leading Brazilian medical societies, addresses this critical need by providing evidence-based strategies for the treatment of obesity and prevention of CVD. Importantly, the guideline considers the specific public health context of the Brazilian population, offering recommendations that carefully considers the risks and benefits of each therapeutic approach.

We acknowledge that implementing these recommendations for the Brazilian population presents a great challenge. Given there are currently no anti-obesity pharmacological therapies available in the Unified Health System (SUS), it is essential to prioritize interventions with proven efficacy to reduce cardiovascular events in the groups with highest CV risk. This approach may support policymakers in cost-effective resource plans to control obesity and reduce associated complications.

## Supplementary Information

Additional file 1

## Data Availability

No datasets were generated or analysed during the current study.

## Abbreviations

ASCVD: Atherosclerotic cardiovascular disease
BMI: Body mass index
CAD: Coronary artery disease
Capacity: Exercise capacity
CKD: Chronic kidney disease
CVD: Cardiovascular disease
GLP-1 RA: GLP-1 receptor agonist
GLP-1/GIP RA: Dual GLP-1/GIP receptor agonist
HF: Heart failure
HFpEF: Heart failure with preserved ejection fraction
HFrEF: Heart failure with reduced ejection fraction
LSM: Lifestyle modification
MACE: Major adverse cardiovascular events (CV death, non-fatal MI, and non-fatal stroke)
NYHA: New york heart association
OSA: Obstructive sleep apnoea
T2D: Type 2 Diabetes
SELECT: Semaglutide effects on cardiovascular outcomes in individuals with overweight or obesity
AF: Atrial fibrillation
SCALE: Satiety and clinical adiposity–Liraglutide evidence
DPP: Diabetes prevention programme
NT-proBNP: N-terminal pro-B-type natriuretic peptide
BNP: B-type natriuretic peptide
AU: Agatston units
eGFR: Estimated glomerular filtration rate
