# Supplementary material for: 2025 Brazilian evidence-based guideline on the management of obesity and prevention of cardiovascular disease and obesity-associated complications: a position statement by five medical societies
Source: Diabetol Metab Syndr. 2025 Nov 18;17:432. doi: 10.1186/s13098-025-01954-8 (PMC12625227; doi:10.1186/s13098-025-01954-8)
Supplement: Supplementary file 1 — Additional file 1 [file 13098_2025_1954_MOESM1_ESM.docx]

**Description of data:**

| SCREENING FOR RISK FACTORS FOR ASCVD AND HF | |
| --- | --- |
| **Hypertension**  For the screening and diagnosis of hypertension in adults, a systolic pressure ≥ 140 mmHg and/or diastolic ≥ **90** mmHg, confirmed in two or more measurements on separate occasions should be used as the diagnostic criterion. Individuals with systolic pressure between 130-139 mmHg and/or diastolic pressure **85**-**89** mmHg should be considered as having pre-hypertension.  **Dyslipidemia**  Universal screening for hypercholesterolaemia is recommended starting at 9 and. In cases with a family history of hypercholesterolaemia, screening should begin after age of **2**. Assessment should include measurements of both cholesterol and triglyceride levels.  **Type 2 diabetes**  All individuals who are overweight or obese should be screened for type 2 diabetes beginning at age 35, using both fasting blood glucose and HbA1c tests. Before the age of 35, screening is mandatory if at least one of the additional risk factors for diabetes is present (see Table 1). Screening may also be considered before the age of 35, on a case-by-case basis, at the discretion of the healthcare provider and in consultation with the patient. | |
| **TABLE 1. RISK FACTORS THAT INDICATE SCREENING FOR TYPE 2 DIABETES IN PATIENTS WITH OVERWEIGHT OR OBESITY** |  |
| - 1. Previously elevated fasting glucose (>100 mg/dL).   2. Previous gestational diabetes.   3. Mother of a large-for-gestational-age newborn.   4. Family history of type 2 diabetes in a first-degree relative.   5. History of clinical CVD (ACS/AMI, chronic CAD, ischemic stroke or TIA, arterial revascularisation), or subclinical CVD (CAC >0 or presence of plaque in the carotid artery).   6. Presence of hypertension, treated or not.   7. HDL <35 mg/dL.   8. Triglycerides > 250 mg/dL.   9. Polycystic ovary syndrome.   10. Presence of acanthosis nigricans.   11. Sedentary lifestyle.   12. Metabolic steatotic liver disease (MASLD or MASH).   13. FINDRISC high or very high. |  |
